# Endocannabinoid tone is higher in healthy lean South Asian than white Caucasian men

**DOI:** 10.1038/s41598-017-07980-5

**Published:** 2017-08-08

**Authors:** Vasudev Kantae, Kimberly J. Nahon, Maaike E. Straat, Leontine E. H. Bakker, Amy C. Harms, Mario van der Stelt, Thomas Hankemeier, Ingrid M. Jazet, Mariëtte R. Boon, Patrick C. N. Rensen

**Affiliations:** 1 Division of Analytical Biosciences, Systems Pharmacology Cluster, Leiden Academic Centre for Drug Research, Leiden University, Einsteinweg 55, Leiden, 2333 CC The Netherlands; 2 Department of Medicine, Division of Endocrinology, Leiden University Medical Center, Albinusdreef 2, Leiden, 2333 ZA The Netherlands; 3 Einthoven Laboratory for Experimental Vascular Medicine, Leiden University Medical Center, Albinusdreef 2, Leiden, 2333 ZA The Netherlands; 4 Department of Molecular Physiology, Leiden Institute of Chemistry, Leiden University, Einsteinweg 55, Leiden, 2333 CC The Netherlands

## Abstract

South Asians have a higher risk to develop obesity and related disorders compared to white Caucasians. This is likely in part due to their lower resting energy expenditure (REE) as related with less energy-combusting brown adipose tissue (BAT). Since overactivation of the endocannabinoid system is associated with obesity and low BAT activity, we hypothesized that South Asians have a higher endocannabinoid tone. Healthy lean white Caucasian (n = 10) and South Asian (n = 10) men were cold-exposed to activate BAT. Before and after cooling, REE was assessed and plasma was collected for analysis of endocannabinoids and lipids. At thermoneutrality, South Asians had higher plasma levels of 2-arachidonoylglycerol (2-AG; 11.36 vs 8.19 pmol/mL, p < 0.05), N-arachidonylethanolamine (AEA; 1.04 vs 0.89 pmol/mL, p = 0.05) and arachidonic acid (AA; 23.24 vs 18.22 nmol/mL, p < 0.001). After pooling of both ethnicities, plasma 2-AG but not AEA positively correlated with triglycerides (R^2^ = 0.32, p < 0.05) and body fat percentage (R^2^ = 0.18, p < 0.05). Interestingly, AA negative correlated with REE (R^2^ = 0.46, p < 0.001) and positively with body fat percentage (R^2^ = 0.33, p < 0.01). Cooling increased endocannabinoids. In conclusion, South Asian compared to white Caucasian men have higher endocannabinoid tone. This suggests that endocannabinoids may, at least in part, underlie the disadvantageous metabolic phenotype of South Asians later in life.

## Introduction

South Asians originally descend from the sub-Indian continent and comprise about 24% of the world population. This population is at higher risk for developing a disadvantageous metabolic phenotype consisting of obesity, dyslipidemia and insulin resistance compared to white Caucasians, making them more prone to develop type 2 diabetes (T2D) at a younger age and lower body mass index (BMI)^1^. The underlying mechanism of the increased predisposition for this unfavorable metabolic profile in South Asians is not well understood, but might be related to a disturbed energy metabolism^2^.

The endocannabinoid system (ECS) is known to play an important role in energy metabolism by regulating appetite, lipolysis and energy expenditure^3^. The ECS is composed of endogenous lipid messengers (endocannabinoids), two distinct G-protein-coupled receptors, i.e. type 1 and type 2 cannabinoid (CB_1_ and CB_2_) receptors, and enzymes responsible for the synthesis and inactivation of the endocannabinoids. *N*-arachidonylethanolamine (anandamide; AEA) and 2-arachidonoylglycerol (2-AG) are the best-studied endocannabinoids. They are synthesized on demand from the membrane lipid precursors *N*-acylphosphatidylethanoamines and diacylglycerides, respectively. Furthermore, there are endogenous bioactive lipids called *N*-acylethanolamines (NAEs), such as *N*-linoleoylethanolamine (LEA), *N*-palmitoylethanolamine (PEA), *N*-oleoylethanolamine (OEA) and *N*-stearoylethanolamine (SEA), which are produced through the same biosynthetic pathway as AEA. NAEs are able to indirectly modulate cannabinoid receptor activity by interfering with endocannabinoid metabolism. In addition, OEA modulates satiety through its interaction with PPARα^4, 5^. The ECS is present in both central and peripheral tissues that are involved in maintaining energy balance. These include the hypothalamus, liver, pancreas, skeletal muscle, white adipose tissue (WAT) and brown adipose tissue (BAT)^6^.

High ECS activity has been associated with human obesity^7, 8^. More specifically, elevated circulating AEA and 2-AG levels have been reported in obese individuals^8–10^ and circulating 2-AG levels are positively correlated with different measures of adiposity, including BMI and body fat percentage^8^, supporting a causal role of the ECS in energy metabolism. Indeed, reduction in 2-AG formation has been associated with reduced food intake in fasted mice^11, 12^ and chronic systemic blockade of the CB_1_ receptor with the inverse agonist rimonabant leads to long-term maintained weight loss and reduction of dyslipidemia in obese rodents^13, 14^ and humans^15–17^.

Mouse studies have shown that the beneficial metabolic effects of CB_1_ receptor blockade are, at least in part, mediated via activation of energy-combusting BAT^18^. Moreover, cold mediated BAT activation leads to a tissue-specific upregulation of endocannabinoids in BAT via CB_1_ receptors, thereby possibly controlling BAT activity by negative feed-back mechanisms^19^. BAT is present and active in human adults and dissipates triglyceride (TG)-derived fatty acids towards heat, thereby contributing to energy expenditure^20–23^. Cold acclimatization, the most important physiological activator of BAT, has been shown to recruit and activate BAT in obese humans^24^. The precise role of the ECS in human BAT activation remains to be determined. Interestingly, we have previously shown that South Asian individuals have lower BAT volume and activity, as measured with [^18^F]fluorodeoxyglucose ([^18^F]FDG) PET-CT scanning, compared to white Caucasians, which might, at least in part, contribute to their high susceptibility to develop obesity and T2D^25^. The underlying cause of the decreased BAT volume in South Asians is still a question that remains.

The main aim of this study was to investigate if healthy lean South Asians men who have not yet developed a disadvantageous metabolic phenotype have a higher endocannabinoid tone as compared to white Caucasians. Furthermore, we investigated whether cold mediated BAT activation leads to upregulation of endocannabinoid levels in humans. In addition, we aimed to assess whether plasma endocannabinoid levels correlate with BAT function, energy expenditure and serum lipid levels.

## Results

### Clinical characteristics

The characteristics of the participants are partly previously described in ref. 25. Twenty-four healthy lean men were included, however, sufficient plasma to analyse the endocannabinoids of only twenty participants (10 white Caucasians and 10 South Asians) was available. In this cohort of 20 participants, mean age was comparable between South Asians and white Caucasians (23.7 vs 25.2 years, respectively) as was BMI (21.4 vs 22.3 kg/m^2^), body fat percentage (23.4 vs 19.4%) and serum TG concentration (0.91 vs 0.82 mmol/L). South Asians had lower REE during thermoneutral conditions (1304 vs 1671 kcal/day, p < 0.01) and cold conditions (1480 vs 2063 kcal/day, p < 0.01), also after correction for lean body mass. Cold exposure increased REE significantly only in white Caucasians (+23.4%, p < 0.01). Furthermore, BAT volume was lower in South Asians as compared to white Caucasians (185 mL vs 303 mL), which was borderline significant (p = 0.052) while the difference was significant in the original study^25^ due to larger sample size.

### Circulating endocannabinoid levels are higher in South Asians and are increased after cooling

**Figure 1:**
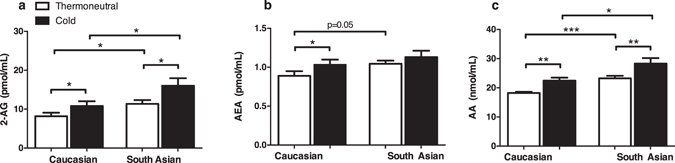
Circulating endocannabinoid levels are higher in South Asians and increased after short-term cooling. Blood was collected from healthy young South Asian (n = 10) and matched white Caucasian (n = 10) individuals before (thermoneutral) and after short-term mild cooling (Cold). Liquid chromatography coupled with tandem mass spectrometry (LC-MS/MS) was used to measure plasma concentrations of 2-AG (**a**), AEA (**b**), and AA (**c**) concentrations. Values are mean ± SEM. *P < 0.05, **P < 0.01, ***P < 0.001. P-values are based on paired t-tests (effect of cold) or unpaired t-tests (effect of ethnicity).

At thermoneutrality, South Asians had higher mean levels of plasma 2-AG compared to white Caucasians (11.36 vs 8.19 pmol/mL, p < 0.05) (Fig. 1a). In addition, there was a trend towards higher mean levels of AEA in South Asians (1.04 vs 0.89 pmol/mL, p = 0.05) (Fig. 1b). Levels of AA were also higher in South Asians (23.24 vs 18.22 nmol/mL, p < 0.001) (Fig. 1c). No significant differences were observed for other NAEs and mono- and di-acyl glycerols measured, except LEA (*N*-linoleoylethanolamide), of which mean plasma levels were higher in South Asians compared to white Caucasians (5.88 vs 3.99 pmol/mL, p < 0.005) (Supplementary Table S1). Collectively, these results suggest that circulating endocannabinoid levels (2-AG and AEA) are higher in lean healthy male South Asians as compared to white Caucasians. We next assessed the endocannabinoid levels in plasma samples collected after short-term mild cold exposure. Interestingly, after cooling circulating levels of 2-AG were higher in South Asians (+41%, p < 0.05) as well as white Caucasians (+32%, p < 0.05) (Fig. 1a). Furthermore, AEA levels were significantly elevated after mild cold exposure in white Caucasians only (+16%, p < 0.05) (Fig. 1b). Plasma AA significantly increased after cold exposure in both ethnic groups (South Asians (+22%, p < 0.01) and white Caucasians (+23%, p < 0.01) (Fig. 1c). The relative increases in endocannabinoid levels upon cooling were not significantly different between the two ethnicities.

### Circulating endocannabinoid levels correlate with serum triglycerides and body fat

**Figure 2:**
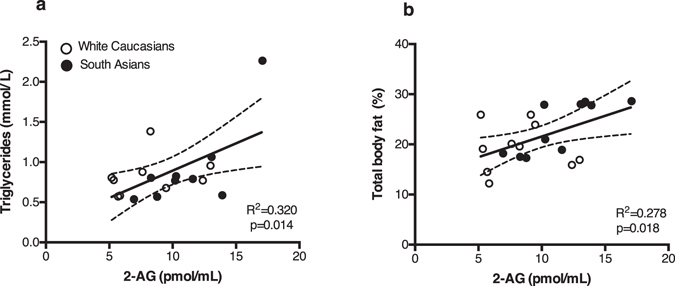
Thermoneutral plasma 2-AG levels positively correlate with serum triglyceride levels and total body fat percentage. Scatterplot of the correlations between 2-AG levels and serum TG (**a**) or total body fat percentage (**b**). Correlations are shown for the total group combined (n = 20), white circles are white Caucasian individuals (n = 10) and black circles are South Asian individuals (n = 10), with 95% confidence limits. Correlations were analysed using linear regression analysis.

**Figure 3:**
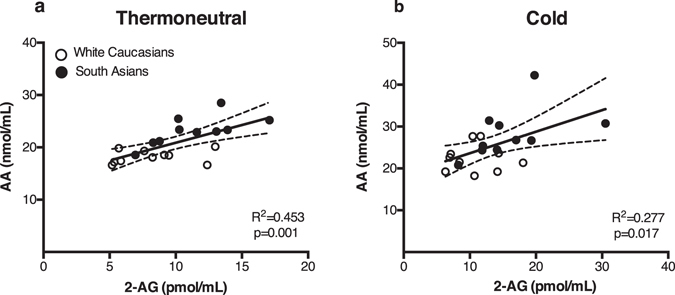
Plasma 2-AG levels correlate with plasma AA levels. Scatterplot of the correlations between plasma 2-AG and AA in both Caucasian and South Asian individuals (n = 40). Correlation is shown for the total group combined under thermoneutral (**a**) and cold conditions (**b**), black circles are South Asian individuals and white circles are white Caucasian individuals, with 95% confidence limits. Correlations were analysed using linear regression analysis.

**Figure 4:**
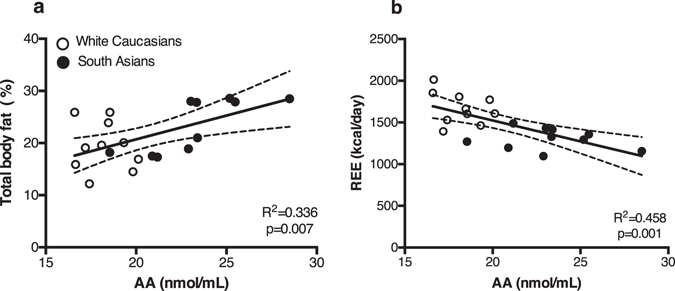
Thermoneutral plasma AA levels positively correlate with body fat percentage and negatively correlate with resting energy expenditure. Scatterplot of the correlations between plasma AA levels measured at thermoneutrality and total body fat percentage (**a**) or thermoneutral resting energy expenditure (REE) (**b**) (n = 20). Correlations are shown for the total group combined, black circles are South Asian individuals and white circles are white Caucasian individuals, with 95% confidence limits. Correlations were analysed using linear regression analysis.

As the ECS is known to play an important role in energy metabolism, we investigated whether circulating endocannabinoid levels correlated with different metabolic parameters in our study cohort. For these analyses we used combined data of South Asians and white Caucasians. Thermoneutral 2-AG levels positively correlated with TG levels (R^2^ = 0.32, p < 0.05) (Fig. 2a) and total body fat percentage (R^2^ = 0.27, p < 0.05) (Fig. 2b), but not with visceral fat percentage (data not shown). In contrast, AEA levels did not correlate with any of these metabolic parameters (data not shown). Additionally, 2-AG positively correlated with AA under thermoneutral (R^2^ = 0.45, p = 0.001) and cold conditions (R^2^ = 0.28 p < 0.05) (Fig. 3). AA also positively correlated with body fat percentage (R^2^ = 0.34, p < 0.01) (Fig. 4a). Interestingly, a strong negative correlation was observed with AA and REE (R^2^ = 0.46, p < 0.001) (Fig. 4b), with AA levels being higher in South Asians compared to white Caucasians (23.24 vs 18.22 nmol/mL, p < 0.001). AA did not correlate with either fat or glucose oxidation (data not shown). We found no significant correlations between circulating endocannabinoids and other metabolic parameters, including (systolic and diastolic) blood pressure, heart rate, fasting serum glucose or C-reactive protein (CRP) levels (data not shown). After cold exposure, there was no correlation between circulating endocannabinoids and REE (data not shown). Both thermoneutral and cold-induced REE positively correlated with BAT volume, but not activity (data not shown)^25^. To further understand the inter-relationship between endocannabinoids and BAT metabolism, we performed several regression analyses. Notably, no correlation was found between circulating endocannabinoid levels and BAT volume (2-AG: R^2^ = 0.016, p = 0.611; AEA: R^2^ = 0.024, p = 0.531; and AA: R^2^ = 0.066, p = 0.288) or activity (2-AG: R^2^ = 0.008, p = 0.714; AEA: R^2^ = 0.132, p = 0.126; and AA: R^2^ = 0.031, p = 0.473), nor was there a correlation between cold-induced or delta (cold minus thermoneutral) endocannabinoid levels upon cooling and BAT activity or volume (data not shown). To test whether the effects could be attributed to ethnicity, we also performed the regression analysis including ethnicity as covariate. Correcting for the effect of ethnicity, plasma 2-AG levels still correlated with serum TG levels (p < 0.05) and tended to correlate with body fat percentage (p = 0.08). In addition, thermoneutral 2-AG levels still correlated with thermoneutral AA levels (p < 0.05), but not under cold conditions (p = 0.12). Also, after correction for ethnicity the negative correlation between AA and REE was not significant anymore (p = 0.41).

## Discussion

The contribution of the ECS in energy metabolism has been well established in humans and research has focused on manipulating this system for the treatment of metabolic disease, such as obesity and T2D. The South Asian population is very prone to develop metabolic disease; however, the underlying mechanism remains to be elusive. In the present study, we showed that healthy lean South Asian men without an apparent disadvantageous metabolic phenotype have higher circulating levels of endocannabinoids (2-AG and AEA) and their metabolite AA compared to matched white Caucasian men. In addition, plasma 2-AG levels positively correlate with serum TG and body fat percentage, and AA levels negatively correlate with REE and positively with body fat percentage.

To the best of our knowledge, this is the first study that shows higher plasma 2-AG, AEA and AA levels in healthy South Asians compared to white Caucasians. Jumpertz *et al*.^26^ previously showed higher 2-AG levels in cerebrospinal fluid (CSF) of American Indians compared to white Caucasians. However, they did not observe differences in 2-AG or AEA levels in plasma of these individuals. This might be due to differences in the study population or a difference in the sensitivity of the assay. For example, Jumpertz *et al*. included young (±29 year), overweight individuals, while in the current study young (±24 year), healthy individuals were included. Furthermore, in South Asians we found higher levels of AA, the breakdown product of endocannabinoids. Although AA can also be synthesized independent of endocannabinoids, these data support the notion that the endocannabinoid tone is higher in healthy young male individuals of South Asian compared to white Caucasian origin.

High circulating endocannabinoid levels in South Asians can result in overstimulation of cannabinoid receptors on tissues such as liver, WAT, skeletal muscle and pancreas. This in turn can lead to several disadvantageous metabolic effects. In mice, a high endocannabinoid tone in the liver, for example, contributes to the development of fatty liver disease^27–29^. In addition, in WAT CB_1_ receptor activation promotes adipogenesis, lipogenesis and energy storage instead of combustion^3^. Moreover, CB receptor stimulation in WAT and skeletal muscle has shown to disrupt insulin signaling thereby promoting insulin resistance, which in combination with reduced insulin secretion from the pancreas (also induced by CB receptor stimulation on pancreatic islet cells) might promote development of T2D^3, 30^. Taken together this indicates that overall high endocannabinoid tone can deteriorate/induce metabolic disease. As South Asians have already been shown to have more hepatic steatosis^31^, adipocyte hypertrophy^31^, disturbed muscle insulin signaling^2^ and lower REE^25^ (i.e. high endocannabinoid tone reduces REE^18^ and high plasma AA levels negatively correlate with REE (Fig. 4b)) compared to white Caucasians, they are possibly at even greater risk for the negative metabolic effects of high endocannabinoid tone.

In addition, we observed positive correlations between plasma 2-AG and serum TG and body fat percentage, albeit the correlation with TG was enforced by a biological outlier. These results are in line with results from Bluher *et al*.^8^ who showed positive correlations between circulating 2-AG levels and both body fat percentage and serum TG in lean and obese individuals. However, they also showed positive correlations between 2-AG and BMI and free fatty acids which could not be reproduced in our study population^8^. Jumper *et al*.^26^ showed positive correlations between AEA, but not 2-AG, and BMI and body fat percentage. However, they studied overweight individuals whereas we studied healthy lean individuals. Consistent with our data, they also found no correlation between plasma 2-AG or AEA levels and REE^26^.

Furthermore, we found that plasma 2-AG and AA levels in both ethnicities are increased after short-term mild cooling, the physiological stimulator of BAT^32^. This observation is consistent with a study in mice showing that BAT activation increases expression and protein levels of endocannabinoids in BAT^19^. The authors hypothesized that increase in endocannabinoids in BAT represents a negative feedback mechanism, as they could locally act on the presynaptic nerve terminal to inhibit noradrenalin release, thereby preventing excessive activation of BAT^19^. Possibly, also in humans the increase in 2-AG and AEA could be derived from BAT, however in our observational study the contribution of other tissues to the plasma endocannabinoid levels could not be excluded. Another explanation for the increased endocannabinoid levels upon cooling could be a higher availability of biosynthetic precursors such as diacylglycerols (DAGs) or *N*-acyl-phosphatidyl-ethanolamines (NAPEs), which are generated during lipolysis of WAT and/or BAT during cooling^19^. In addition, it has been shown that cold exposure reduces the mRNA expression of the breakdown enzyme *FAAH* in BAT, which might also in part explain higher plasma endocannabinoid levels upon cooling^19^. Alternative explanations can also not be excluded at present. For example, 2-AG has a circadian rhythm in humans with the lowest plasma concentration peak around 5:00 am and the highest peak concentration around 13:00 pm^33^. Since the blood samples in the current study were withdrawn around 9:30 (thermoneutrality) and 12:30 (cold), a small increase, of approx. 15%, in plasma 2-AG could already be expected from its circadian rhythm. However, we found a larger 37% average increase in 2-AG when combining the South Asians and white Caucasians. In addition, cooling elicits a stress response in the body, and stress, via glucocorticoids, has been shown to increase the peripheral ECS tone^34, 35^.

Previous mouse studies showed that global blockade of the CB_1_ receptor by rimonabant activates BAT, markedly enhances energy expenditure and leads to a tissue-specific upregulation of endocannabinoids^18, 19^. In fact, human studies showed that taranabant, another inverse agonist of the CB_1_ receptor also reduced food intake, induced weight loss and increased REE and fat oxidation in obese individuals^36^. Therefore, we hypothesized that these effects may have been due to BAT activation. However, in our study there was no significant correlation between plasma endocannabinoids and BAT volume/activity, fatty acid or glucose oxidation. Possibly our sample size was too small to reliably assess this, as the variation in BAT between subjects was rather large.

A strength of this study is that we investigate differences in circulating endocannabinoid levels in lean healthy South Asians and white Caucasians individuals before they develop metabolic diseases. This allowed us to investigate in more detail whether ethnic differences in endocannabinoid tone are already present before the onset of the disease rather than being a consequence of the metabolic disease. However, a limitation of our study is that we could only measure circulating endocannabinoid levels which do not necessarily reflect endocannabinoid signalling within peripheral organs and thus the endocannabinoid function. For future studies, it would be interesting to measure endocannabinoid levels and levels of endocannabinoid synthesis and breakdown enzymes in specific tissues, such as WAT and skeletal muscle. In addition, we cannot exclude that ‘thermoneutrality’ might have been experienced differently between the two ethnicities, which might have biased our results. Furthermore, the small sample size might have limited the number of correlations we could detect. However, despite this we were able to reproduce some correlations shown previously by others underscoring the robustness of these findings. Future studies in larger cohorts should verify the translational value of these results for the general population.

In conclusion, our data demonstrate that healthy lean South Asian men have a higher endocannabinoid tone compared to white Caucasian men. Provided that our cohort is representative for the general population, this might, at least in part, contribute to the development of a disadvantageous metabolic phenotype later in life. These results are in line with the hypothesis that the ECS functions as a negative feedback system on BAT. Future research should focus on elucidating the underlying cause of the high endocannabinoid tone in this vulnerable population and verify potential causal relationships. This knowledge might lead to the development of novel treatment strategies to combat metabolic disease.

## Materials and Methods

### Ethics

Venous blood samples were collected as part of a previously conducted observational study aimed at investigating BAT activity and volume in Dutch South Asian and white Caucasian individuals^25^. This study was approved by the Medical Ethical Committee of the Leiden University Medical Center (LUMC) and undertaken in accordance with the principles of the revised Declaration of Helsinki. All volunteers provided written informed consent. Trial registration number and date: Netherlands Trial Register 2473 and 13-08-2010.

### Participants and study design

We enrolled twenty-four Dutch healthy lean men, between 18–28 years of age, with a BMI 18–25 kg/m^2^. Twelve men were of Dutch South Asian descent and twelve of Dutch white Caucasian descent^25^. This study was conducted at the Alrijne Hospital, Leiderdorp (the Netherlands) between March 2013 and June 2013^25^. In short, subjects underwent medical screening including their medical history, a physical examination, blood chemistry tests and an oral glucose tolerance test to exclude individuals with type 2 diabetes according to the American Diabetes Association 2010 criteria. Other exclusion criteria were rigorous exercise, smoking and recent body weight change up to 3 months prior to the start of the study.

### Study procedures

Subjects were studied after overnight fasting and 24 hours without heavy exercise. We determined body composition using a Dual Energy X-ray Absorptiometry (DEXA) scan (iDXA, GE healthcare, UK). Next, an intravenous cannula was inserted in the antecubital vein for blood collection and injection of [^18^F]FDG. To assess BAT volume and activity, subjects were exposed to an individualized water cooling protocol to maximally activate BAT. In short, subjects were cooled for approximately 2 hours until just above shivering temperature, to ensure maximum non-shivering thermogenesis, followed by an [^18^F]FDG PET-CT scan (Gemini TF PET-CT, Philips Healthcare, Best, the Netherlands). For the individual cooling protocol, subjects lay between two water perfused cooling mattresses (Blanketrol III, Cincinnati Sub-Zero Products, Cincinnati, OH, USA). After 60 minutes at 32 °C (thermoneutral temperature), the temperature of the mattresses were gradually decreased with 5 °C every 10 minutes until shivering occurred. At this point, the temperature was raised by 3 °C and the official cooling period of 2 hours was started. After 1 hour of cooling, 2 MBq/kg [^18^F]FDG was administrated intravenously. Both in thermoneutral and cold-induced condition, indirect calorimetry was performed with a ventilated hood (Oxycon Pro™, CareFusion, Heidelberg, Germany). Venous blood samples were collected in a fasted state at the end of the thermoneutral period and at the end of the cooling period. After 2 hours of cooling, an [^18^F]FDG PET-CT scan was acquired starting with a low-dose CT scan followed by PET scanning.

### Brown adipose tissue measurements and energy expenditure

BAT volume (in mL) and BAT activity (in standardized uptake value (SUV)) in the region of interest were determined from the [^18^F]FDG PET-CT scans (Gemini TF PET CT, Philips, Best, the Netherlands) by a blinded nuclear physician and a researcher. Resting energy expenditure (REE), respiratory quotient and rates of lipid and glucose oxidation were measured with indirect calorimetry using a ventilated hood system (Oxycon Pro, CareFusion, Heidelberg, Germany).

### Serum lipid measurements

Serum TG and free fatty acid concentrations were determined in the blood samples with the use of commercially available enzymatic kits 11488872 and 91096 (Roche Molecular Biochemicals, Indianapolis, USA) as described previously^25^.

### Endocannabinoid measurements

Plasma sample of only 20 subjects could be studied since four subjects were excluded due to insufficient volume of plasma sample left available for the analysis in 2 individuals of each ethnicity. In total, endocannabinoids and their congeners were quantified in 40 plasma samples using liquid chromatography coupled with tandem mass spectrometry (LC-MS/MS). From the pool of individual study samples, quality controls (QCs) were used to generate calibration curves. Additionally, all samples were randomized and each batch included calibration samples and an even distribution of QC samples and blanks.

#### Endocannabinoid sample extraction

Endocannabinoid extraction was performed on ice. Briefly, 50 µL of plasma was transferred into 1.5 mL Eppendorf tubes, spiked with 10 µL of deuterated internal standard mix (Supplementary Table S1) followed by addition of 50 µL of 0.1 M ammonium acetate buffer (pH 4). After two times extraction with 400 µL of methyl tert-butyl ether (MTBE) (443808, Sigma Aldrich, Zwijndrecht, the Netherlands), the tubes were thoroughly mixed for 4 min using a bullet blender (Next Advance, Inc., Averill park, NY, USA) at medium speed, followed by a centrifugation step (4 °C, 5,000 g, 12 min). Then 750 μL (combined from two extractions) of the upper MTBE layer was transferred into clean 1.5 mL Eppendorf tubes. Samples were dried in a speed vac followed by reconstitution with 50 μL of acetonitrile/water (90/10, v/v). The reconstituted samples were centrifuged (4 °C, 14,000 g, 3 min) before transferring into LC-MS vials. 5 µL of each sample was injected into the LC-MS/MS system.

#### LC-MS/MS Analysis

A targeted analysis of 22 compounds, including endocannabinoids and related *N*-acylethanolamines (NAEs) along with their precursor molecule and metabolite arachidonic acid (AA) (Supplementary Table S1), was measured using an Acquity UPLC I class Binary solvent manager pump (Waters, Milford, USA) in conjugation with AB SCIEX 6500 quadrupole-ion trap (QTRAP) (AB Sciex, Massachusetts, USA). The separation was performed with an Acquity HSS T3 column (1.2 × 100 mm, 1.8 µm) maintained at 45 °C. The aqueous mobile phase A consisted of 2 mM ammonium formate and 10 mM formic acid, and the organic mobile phase B was acetonitrile. The flow rate was set to 0.4 mL/min; initial gradient conditions were 55% B held for 2 minutes and linearly ramped to 100% B over 6 minutes and held for 2 minutes; after 10 seconds the system returned to initial conditions and held 2 minutes before next injection. Electrospray ionization-MS and a selective Multiple Reaction Mode (MRM) was used for endocannabinoid quantification. Individually optimized MRM transitions using their synthetic standards for target compounds and internal standards are described in Supplementary Table S1. Relative standard deviations (RSD) of QC samples were calculated to monitor the data quality and RSD less than 15% was observed for 90% the target compounds. Peak area integration was performed with MultiQuant (AB Sciex, Version 3.0.2) data analysis software. The obtained peak areas of targets were corrected by appropriate internal standards. Calculated response ratios, determined as the peak area ratios of the target analyte to the respective internal standard, were used to obtain absolute concentrations from their respective calibration curves. The response ratios were further corrected using in-house QC tool^37^.

### Statistical analysis

As previously mentioned, four participants (2 white Caucasians and 2 South Asians) were excluded from the analyses due to insufficient sample for the endocannabinoid measurements. Data were collected and analysed using IBM SPSS statistics version 23.0. Baseline characteristics were compared using unpaired student t-tests. Differences in plasma endocannabinoid levels in (between) groups were analysed using two tailed paired (unpaired) t-tests. Furthermore, linear regression analysis computed by Pearson’s correlation was used to determine correlations between plasma endocannabinoid levels and different metabolic parameters. Regression analysis was performed both with and without correction for the effect of ethnicity, by respectively including/excluding ethnicity as a covariate. P values < 0.05 were considered as statistical significant. Data are presented as mean ± SEM.

## Electronic supplementary material

Supplementary table S1:
