# Supplementary material for: Endocannabinoid tone is higher in healthy lean South Asian than white Caucasian men
Source: Sci Rep. 2017 Aug 8;7:7558. doi: 10.1038/s41598-017-07980-5 (PMC5548787; doi:10.1038/s41598-017-07980-5)
Supplement: Supplementary file 1 — Supplementary table S1: [file 41598_2017_7980_MOESM1_ESM.doc]

**Supplementary Information**

**Endocannabinoid tone is higher in healthy lean South Asian than white Caucasian men**

Vasudev Kantae^1,*,+^, Kimberly J. Nahon^2,3,+^, Maaike E. Straat^2,3^, Leontine E.H. Bakker^2^, Amy C. Harms^1^, Mario van der Stelt^4^, Thomas Hankemeier^1^, Ingrid M. Jazet^2,3^, Mariëtte R. Boon^2,3^ and Patrick C.N. Rensen^2,3^

Supplementary table S1:

| **S.No** | **ID** | **Metabolite Name** | **Q1** | **Q3** | **DP, CE** | **Polarity** | **Concentration (pmol/mL)** | | |
| --- | --- | --- | --- | --- | --- | --- | --- | --- | --- |
| *Standards* | | | | | | | **Caucasians** | **South Asians** | **P-Value** |
| 1 | 1-1OG (18:1) | 1-Oleoyl Glycerol | 357 | 265 | 80, 20 | + | 1055±100 | 954±135 | 0.554 |
| 2 | 2-OG (18:1) | 2-Oleoyl Glycerol | 357 | 265 | 80, 20 | + | 97.6±7.2 | 83.0±9.9 | 0.247 |
| 3 | 1-LG (18:2) | 1-Linoleoyl Glycerol | 355 | 263 | 80, 15 | + | 338.0±39.4 | 436.4±54.3 | 0.160 |
| 4 | 2-LG (18:2) | 2-Linoleoyl Glycerol | 355 | 263 | 80, 20 | + | 59.6±8.1 | 74.5±9.3 | 0.244 |
| 5 | 2&1-AG (20:4) | 2&1-Arachidonoylglycerol | 379 | 287 | 56,19 | + | 8.19±0.89 | 11.36±0.97 | 0.028^*^ |
| 6 | 2AGE (20:4) | 2-Arachidonyl Glycerol ether | 365 | 273 | 72, 25 | + | ND | ND | - |
| 7 | PDEA (15:0) | *N*-Pentadecanoylethanolamide | 286 | 62 | 80, 18 | + | 0.23±0.13 | 0.10±0.01 | 0.312 |
| 8 | PEA (16:0) | *N*-Palmitoylethanolamide | 300 | 62 | 78, 36 | + | 9.28±0.71 | 10.60±0.26 | 0.099 |
| 9 | SEA (18:0) | *N*-Stearoylethanolamide | 328 | 62 | 72, 31 | + | 7.24±0.33 | 7.41±0.19 | 0.677 |
| 10 | POEA (16:1) | *N*-Palmitoleoylethanolamide | 298 | 62 | 86, 32 | + | 1.03±0.15 | 1.02±0.12 | 0.990 |
| 11 | OEA (18:1) | *N*-Oleoylethanolamide | 326 | 62 | 72, 30 | + | 6.57±0.43 | 7.39±0.38 | 0.175 |
| 12 | LEA (18:2) | *N*-Linoleoylethanolamide | 324 | 62 | 74, 36 | + | 3.99±0.39 | 5.88±0.39 | 0.003^*^ |
| 13 | Alpha-LEA (18:2) | *N*-α-Linolenylethanolamide | 322 | 62 | 80, 20 | + | 0.01±0.01 | 0.01±0.01 | 0.408 |
| 14 | DGLEA (18:3) | Dihomo-γ-Linolenoyl Ethanolamide | 350 | 62 | 70, 20 | + | 0.21±0.02 | 0.20±0.02 | 0.796 |
| 15 | ETAEA (20:3) | Eicosatrienoic Acid Ethanolamide | 350 | 62 | 80, 46 | + | ND | ND | - |
| 16 | AEA (20:4) | Anandamide | 348 | 62 | 70, 42 | + | 0.89±0.06 | 1.04±0.04 | 0.050 |
| 17 | O-AEA (20:4) | O-Arachidonoyl ethanolamine | 348 | 62 | 70, 25 | + | ND | ND | - |
| 18 | EPEA (20:5) | Eicosapentaenoyl Ethanolamide | 346 | 62 | 80, 20 | + | 0.05±0.01 | 0.07±0.01 | 0.191 |
| 19 | DEA (22:4) | *N*-Docosatetraenoylethanolamide | 376 | 62 | 72, 20 | + | 0.27±0.02 | 0.28±0.03 | 0.916 |
| 20 | DHEA (22:6) | *N*-Docosahexaenoylethanolamide | 372 | 62 | 70, 25 | + | 5.14±0.44 | 5.61±0.55 | 0.522 |
| 21 | NADA (28:4) | *N*-Arachidonoyl dopamine | 440 | 137 | 75, 24 | + | ND | ND | - |
| 22 | Arachadonic Acid (20:4) | 5,8,11,14-Eicosatetraenoic acid | 303 | 259 | -80, -18 | - | 18223±401 | 23244±872 | 0.001^***^ |
| *Internal standards* | | | | | | | | | |
| 1 | 2-AG (20:4)-d8 | 2-Arachidonoylglycerol-d8 | 387 | 294 | 56, 20 | + | - | - | - |
| 2 | PEA (16:0)-d4 | Palmitoyl ethanolamide-d4 | 304 | 62 | 78, 36 | + | - | - | - |
| 3 | SEA (18:0)-d3 | Stearoyl ethanolamide-d3 | 331 | 62 | 72, 31 | + | - | - | - |
| 4 | OEA (18:1)-d4 | Oleoyl Ethanolamide-d4 | 330 | 66 | 72, 30 | + | - | - | - |
| 5 | LEA (18:2)-d4 | Linoleoyl ethanolamide-d4 | 328 | 66 | 74, 36 | + | - | - | - |
| 6 | AEA (20:4)-d8 | Arachidonoyl Ethanolamide-d8 | 356 | 62 | 70, 42 | + | - | - | - |
| 7 | DHEA (22:6)-d4 | Docosahexaenoyl Ethanolamide-d4 | 376 | 66 | 70, 25 | + | - | - | - |
| 8 | NADA (28:4)-d8 | *N*-Arachidonoyl dopamine-d8 | 448 | 137 | 75, 34 | + | - | - | - |

Supplementary Table S1: MRM (Multiple reaction monitoring) parameters and endogenous concentrations of the target list

The target list includes endocannabinoids and *N*-acylethanolamines (NAEs) and deuterated (d) labelled internal standards. The compound ID is the abbreviation of metabolite name along with number of carbon atoms and number of double bonds in the fatty acid chain of the molecule, respectively. All compounds are analysed in positive mode except arachidonic acid in negative mode. Q1 and Q3 are optimized precursor ion and product ion, expressed as *m/z*. DP and CE are declustering potential (Volts) and collision energy (volts). Endogenous concentrations are denoted as mean ± SEM (standard error of mean). *P<0.05, ***P<0.001. P-values are based on unpaired t-tests. ND: Not detected in human plasma.
